# Industry–academia interface: how to build industry-university partnerships with Mark Jefferies

**DOI:** 10.1038/s44172-026-00683-8

**Published:** 2026-05-15

## Abstract

Mark Jefferies is Head of Research Partnerships at Rolls-Royce. Here Mark shares his views of the factors that lead to successful long-term university-industry collaborations, and the challenges that might be met along the way. We also catch a glimpse of how Rolls-Royce’s research goals are changing in response to sustainability targets and other technology drivers.

**Figure:**
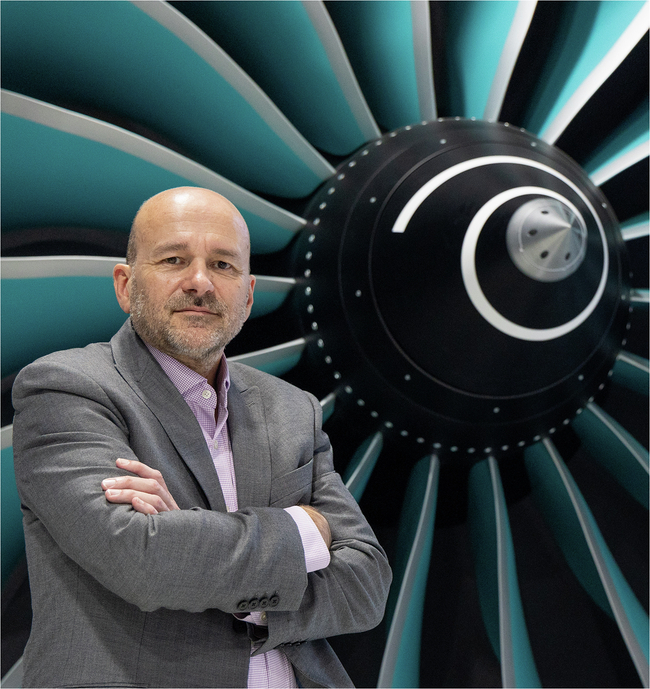
Rolls-Royce Image Resources.

1. Tell us about your current role within Rolls-Royce?

I’ve been involved at the interface of Rolls-Royce’s strategic university partnerships for many years now. This consistency has helped deliver the type of industry-academic partnerships that makes a material difference both to the company, and to the academic community. I find myself involved in just about every aspect of collaboration you can think of, and possibly several you may not have considered! I build new partnerships, try and predict what technology partnerships we might need for the future, engage with Government, mentor new students and academics, and ensure we have an appropriate level of governance around what we do. I also get to see some amazing engineering and science. Oh, and I travel around the world and meet some outstanding people. That last bit, the people, is the key ingredient for successful collaboration.

2. What do you find the most exciting about working at the academia-industry interface? And most challenging?

Sometimes the results of what I’m involved in take time to materialise, but they can make a real difference to people’s lives. Examples include some of the big research projects and infrastructure, such as the creation of the National Centre for Combustion & Aerothermal Technology (Loughborough University) that required multiple people working together for years to deliver, or the formation of Centres for Doctoral Training in partnership with the Engineering & Physical Sciences Research Council. Being able to walk into a facility or meet the students who are benefitting is very rewarding. Challenges come about for similar reasons. It can take a lot of time and effort, and things don’t always work out. But with resilience, often you can still find positive outcomes even if it doesn’t end up as you originally intended. Other challenges stem from unavoidable changes to business or technology needs. Ending a relationship that you have helped build can be hard. It’s important to treat the people involved with respect, try to avoid it being a surprise, and help find ways they can go on to new things. You never know, there might be other opportunities to collaborate in the future.

3. Rolls-Royce form long term large strategic partnerships with universities. Why have you adopted this strategy for a large part of your research and development work?

Yes, we do. Some of the academics I work with now, I’ve worked with for almost three decades. We do shorter pieces of work too, and we work with others. But the bulk of our academic partnerships are grounded in long term engagement. Other companies engage in lots of small projects wherever they find interesting work. There’s no right or wrong way, only the way that that suits your business. We chose differently, being one of the first companies to pioneer a formal long term engagement model, and it has served us well. It allows us to build trust, invest for the long term, find effective ways of transferring technology and people, and develop people with a deep understanding of what is important to our business. But all of this comes with some risk, which is partly where I come in. It would be easy to see how complacency could creep in, for example continuing a strand of research simply because “that’s what we’ve always done”. Effort is required from both sides to avoid that. We must bring in new people who generate new ideas, continually challenge and expose our work to others to test the direction. And we must share strategy, challenges and ambition between the partnering organisations to provoke constructive debate on what the future might look like. This is where new ideas for what you could create start to take shape.

4. How do you connect with researchers to ensure that Rolls-Royce achieves its business goals whilst the researchers you want to work with achieve their academic ambitions?

This is a good question, and something else that comes with time and understanding of what is important to each organisation. If you have a long-term expectation, then undermining your partner for short term gain would be foolish. I’m very conscious of the challenges faced by academics and the universities themselves – obvious examples include ability to publish for the individuals, a successful thesis for a student, or impact case studies (or “REF” returns) for the institution. It is in our long-term interests that an institution is successful – that success brings with it access to talented people, better research, funding, equipment, and so on – so the question becomes what we can do to help or at least minimise any hindrance. And there will be a tension. We don’t expect everything to always go smoothly. But having a strong relationship at multiple levels lets you work through any issues. It helps if both parties choose people who understand the other side of the partnership – we need to pick our teams carefully! It is not a given that just throwing together researchers and engineers will work because on paper it looks a good fit. Multiple factors come into play: are our goals aligned? Do we have a well understood framework of expectations? Is the work supported by the leadership teams? Does it address a need? Are people willing to put in the effort? Once you have the right people, we then create opportunities for them to come together and encourage them to share what they want to achieve. This can be difficult at first. Everyone is understandably cautious when first meeting which is why encouragement from the leadership is required. Done well, this can lead to some powerful conversations and new ideas. But trust needs to be built first.

5. What do you look for when building new partnerships beyond an outstanding research record?

Some clues to this can be found in the previous question. Excellence in research could be considered the entry ticket, but it is by no means the only factor. You could apply some weighting between different factors which changes depending on what you are trying to achieve, or your type of business, but a lot of things spring to mind. Some examples include what are the parties’ approaches to Intellectual Property: can you reach agreement easily? Or are you going to spend the next two years negotiating? Does the ethos of the business partner and the university align? This is especially important if you work in sensitive topic areas. What type of governance is appropriate? Is this work that should take particular note of the NPSA (National Protective Security Authority) Trusted Research Guidelines for example? What additionality can a partner bring? Again this applies in both directions. It’s about much more than funding, it includes equipment, materials, background knowledge, and more. Oftentimes, proximity matters although not always. In the end it’s a balance. And I have seen relationships succeed or (almost!) fail simply because of individual relationships. The people involved need to respect each other and get on. (Did I mention a lot of it is about people?!)

6. I’d like to change tack for a moment and talk about the actual research that your teams are exploring. How have your research priorities changed to support the energy transition and other sustainability issues e.g. in the aerospace sector?

Given that Rolls-Royce has been around for well over 100 years, it’s no surprise that research priorities have changed. But right now, I am seeing things change faster and more significantly than at any time I can remember. And in such a competitive environment if you don’t change with it, then you are quickly going to get left behind. At the moment, the business is going through a multi-year transformation, and we have a clear roadmap focusing on what we need to do, so our research priorities are being guided by our long-term objectives.

Our product portfolio can be broadly described as delivering complex power and propulsion solutions for safety-critical applications in the air, at sea and on land. For many years, emphasis has been on efficiency, and this remains hugely important to us. In short, the best way to save fuel is to not use it in the first place. The sectors in which we operate are simultaneously vital to the modern economy whilst being both technically difficult to decarbonise and safety critical, not to mention often highly regulated in global markets. You can’t just try something out and hope it works when peoples’ lives are at stake. Our number one priority is to keep our colleagues and customers safe. I think this means balancing economic, environmental, social and ethical needs, and working to understand the impact of our business on the world, and the world’s impact on our business. It’s important to recognise the value in listening to a wide range of stakeholders and working with them. Our research reflects a similar broad but considered approach, ranging from working with leading climate scientists to better understand the effects of carbon and non-carbon environmental impact, modelling the potential outcome of various energy scenarios, continuing to develop more efficient products such as increasing the use of lighter materials, testing and certifying all our in-production engine products on Sustainable Aviation Fuel (and understanding what impact this in itself might have), and investigating how and whether hydrogen, electrification, or other power and fuel options can play a part. This can range from component and system testing through to full engine demonstration.

7. How has the Artificial Intelligence (AI) and the data revolution impacted on your research goals?

We have seen very rapid advancements in digital technologies, and this is likely to be only the beginning. We have developed and run some of the world’s largest computational simulations utilising national supercomputer facilities, crucial to how we design and test jet engines, and use data-driven models that allow us to provide market-leading services to our customers for the life of our products. I am certain we will see such complex models applied more routinely in the future. There remain plenty of unknowns – just when and how quantum computing will become practical for instance. But it is imperative for a technology-led business to be embedded in digital capability both to be able to use it, and to understand how it might impact the business. Imagine your competitors being able to design twice or even ten times as fast as you. We’re working hard at this and investing heavily into modernising our systems. Digital is an integral part of the business’s strategy, including a focus on using AI to create competitive advantage and accelerate design, streamline production and boost efficiency. Digital twins are becoming a reality and are increasingly being used in complex engineering applications to monitor, assess, and respond to the real-world experience of a product. These virtual replicas of physical assets, systems, or processes, enable engineers to monitor, simulate, and optimise performance in real-time, and offer potentially significant benefits including reduced costs, improved efficiency, and faster innovation cycles. However, like many advances, they present challenges of initial implementation, complexity, data management and security concerns, not to mention challenges related to model fidelity, uncertainty, and the need for efficient data processing in potentially harsh environments. AI, in my opinion, will undoubtedly change the way nearly everyone works. It can offer insight, change the way people learn, and accelerate the design process as just a few examples. But it requires care: Is the information reliable? Are there unknown biases? Is it open to misuse? and so on. We must think carefully before applying a tool, and then to use it knowingly.

8. What other emerging trends or factors are guiding the research trajectories of Rolls-Royce that you can share?

The pace at which we can progress and adopt research, innovation, and development will be one of the deciding factors in remaining competitive. We are fortunate to have built up a strong network of research partners, so finding ways of increasing the effectiveness of that network by combining their strengths is one of my key goals. We will see a step change in the way we think about skills and training; just about all human knowledge is available at our fingertips, so we now face the challenge – and opportunity – of moving the mindset from learning things to routinely using that knowledge to identify future trends and solutions. Getting all this into the hands of our engineers and designers will be critical, so that will require effective communication and tools, drawing heavily on the digital thread discussed earlier.

9. Finally, if you could introduce one thing to strengthen bridges between academia and industry, leading to greater societal benefit, what would it be?

Just one? Tough call, but I’m going to pick something I have been saying for a long time – increase the porosity between the different groups. What I mean by that is that we should see a greater exchange of people across the boundaries, making it easier to have a mixed career, or at least be able to spend some time working in the others’ environment. This would benefit both the academic and the company teams, injecting fresh ideas and new thinking into the mix and giving people a wider perspective – ultimately increasingly the likelihood that innovative ideas get adopted faster. There are lots of practical reasons why that isn’t as simple as it sounds, perceptions around career progression, employment terms, or even location for example. But often these things are artificial – people created them in the past for good reason, but the world has changed. Let’s look again at what is needed for the future, not stick to the way things ‘have always been done around here’.

This interview was carried out by Rosamund Daw, Chief Editor, *Communications Engineering* in January of 2026. The interview reflects the personal views of Mark Jefferies and not the views of *Rolls-Royce* or *Communications Engineering*.

